# Ticagrelor improves blood viscosity-dependent microcirculatory flow in patients with lower extremity arterial disease: the Hema-kinesis clinical trial

**DOI:** 10.1186/s12933-019-0882-5

**Published:** 2019-06-07

**Authors:** Robert S. Rosenson, Qinzhong Chen, Sherwin D. Najera, Prakash Krishnan, Martin L. Lee, Daniel J. Cho

**Affiliations:** 1 Cardiometabolics Unit, Zena and Michael A. Wiener Cardiovascular Institute, Marie-Josee and Henry R. Kravis Center for Cardiovascular Health, Icahn School of Medicine at Mount Sinai, One Gustav L. Levy Place, Hospital Box 1030, New York, NY 10029 USA; 2 Cardiac Catheterization Laboratory, Mount Sinai Hospital, Icahn School of Medicine at Mount Sinai, One Gustav L. Levy Place, New York, NY 10029 USA; 3 UCLA Fielding School of Public Health, 650 Charles E. Young Drive S., 51-254 CHS, Los Angeles, CA 90095 USA; 4 Rheovector, LLC, King of Prussia, PA 19406-1405 USA

**Keywords:** Lower extremity arterial disease, Microvascular disease, Blood viscosity, Type 2 diabetes, Ticagrelor

## Abstract

**Background:**

Microvascular blood flow (MBF) impairment in patients with lower extremity arterial disease (LEAD) is associated with more severe major adverse limb events (MALE). The contribution of ticagrelor, a P2Y12 antagonist and an adenosine enhancer, on blood viscosity (BV) and BV-dependent MBF in LEAD is unknown. The aim of the trial is to investigate the effects of ticagrelor on BV, and explore the association of BV-dependent MBF in participants with LEAD and type 2 diabetes (T2DM).

**Methods:**

Randomized, double-blind, double-dummy, crossover trial design that compares treatment with aspirin 81 mg/ticagrelor placebo, aspirin 81 mg/ticagrelor 90 mg twice daily and aspirin placebo/ticagrelor 90 mg twice daily on high-shear (300 s^−1^) and low-shear (5 s^−1^) BV, and laser Doppler flowmetry (LDF) in the dorsum of the feet of participants with T2DM.

**Results:**

We randomized 70 (45% female) participants aged (mean ± SD) 72 ± 9 years. The duration of LEAD was 12.3 ± 10.3 years, and 96.9% reported intermittent claudication symptoms. Use of statins was 93% (high-intensity 43%, moderate intensity 49%), renin–angiotensin–aldosterone system inhibitors (75%) and beta-blockers (61%). Treatment with ticagrelor with or without aspirin reduced high-shear BV by 5%, in both cases, while aspirin monotherapy increased high-shear BV by 3.4% (p < 0.0001). Ticagrelor with or without aspirin reduced low-shear BV by 14.2% and 13.9% respectively, while aspirin monotherapy increased low-shear BV by 9.3% (p < 0.0001). The combination of ticagrelor and aspirin increased MBF in the left foot compared to the other two treatments (p = 0.02), but not in the right foot (p = 0.25).

**Conclusions:**

Ticagrelor should be considered in the treatment of microvascular disease in patients with LEAD and T2DM.

*Trial registration* Registration number: NCT02325466, registration date: December 25, 2014

**Electronic supplementary material:**

The online version of this article (10.1186/s12933-019-0882-5) contains supplementary material, which is available to authorized users.

## Background

Lower extremity arterial disease (LEAD) occurs more often in patients with diabetes than in patients without diabetes [1]. Microvascular disease in patients with diabetes and LEAD is associated with more severe major adverse limb events (MALE) [2]. As compared with non-diabetes patients with LEAD, patients with diabetes have higher rates of severe below-the-knee disease, lower limb amputations and critical ischemia resulting in less effective and durable percutaneous and surgical revascularization rates [3–6].

Multiple studies have shown higher blood viscosity values in patients with type 2 diabetes than controls [7]. Elevated blood viscosity is more common in patients with claudication than controls resulting in shorter mean claudication distance [8, 9]. This phenomenon termed “rheological claudication” was reported in about 25% of patients with moderate to severe claudication and blood hyperviscosity. Low shear blood viscosity influences microcirculatory flow in patients with LEAD [10, 11].

Certain pharmacological therapies recommended for the treatment of intermittent claudication in patients with LEAD reduce blood viscosity including clopidogrel [12] and pentoxifylline [13, 14]. In contrast, other commonly used therapies such as cilostazol or ticlopidine improve pain-free walking distance, but do not alter blood rheology [15]. Ticagrelor is potent a P2Y12 receptor antagonist that also inhibits adenosine uptake via the equilibrative nucleoside transporter 1 (ENT1) transporter and increases adenosine concentrations in acute coronary syndrome patients [16, 17]. In addition ticagrelor stimulates the rapid release of adenosine triphosphate from red blood cells in vitro [18]. The administration of ticagrelor increases adenosine-induced coronary blood flow velocity and improves vascular reactivity compared with clopidogrel [19, 20]. Agents that increase adenosine have been shown to lower blood viscosity [21].

The clinical relevance of reducing blood viscosity on microcirculatory perfusion in patients with LEAD remains unknown. The aim of this clinical trial is to investigate the effects of ticagrelor on high-shear and low-shear blood viscosity, and explore the effect of ticagrelor on microvascular blood flow in patients with LEAD and type 2 diabetes.

## Methods

This study was approved by the institutional review board at the Icahn School of Medicine at Mount Sinai. Written informed consent was obtained from all participants.

### Study design

Details of the trial design have been reported previously. Hema-kinesis is a randomized, double-blind, double-dummy, crossover trial design that compares treatment with aspirin 81 mg/ticagrelor placebo, aspirin 81 mg/ticagrelor 90 mg twice daily and aspirin placebo/ticagrelor 90 mg twice daily on high-shear (300 s^−1^) and low-shear (5 s^−1^) blood viscosity (*NCT02325466*) [22]. The inclusion and exclusion criteria are provided in Table 1. Study participants were recruited from the outpatient cardiology practice and the Cardiac Catheterization database at the Mount Sinai Hospital, New York, NY, USA. An exploratory endpoint was measurement of microvascular blood flow in the dorsum of the feet of participants with type 2 diabetes.

**Table 1:** Inclusion and exclusion criteria

Inclusion criteria	Subject is willing to comply with requirements of the study protocol
	Male and female patients ≥ 18 years of age
	Type 2 diabetes mellitus
	Symptomatic or known PAD/claudication
	Symptomatic PAD or Ankle-brachial index ≤ 0.85 or calcified blood vessels with toe-brachial index ≤ 0.6 and/or abnormal post-exercise ankle–brachial index or prior surgical or percutaneous intervention of the peripheral arteries ≥ 12 months previously with a residual stenoses of ≥ 50% in a non-dilated artery
Exclusion criteria	Type I diabetes, poorly controlled diabetes (HbA1c < 8.5%). Newly diagnosed type 2 diabetes (within 6 months of randomization) or laboratory evidence of diabetes during screening (fasting serum glucose ≥ 126 mg/dL [7.0 mmol/L] or HbA1c ≥ 6.5%) without prior diagnosis of diabetes
	Uncontrolled hypertension defined as sitting systolic blood pressure (SBP) > 180 mmHg or diastolic BP (DBP) > 100 mmHg
	NYHA III or IV heart failure, or last known left ventricular ejection fraction (LVEF < 30%)
	Female subject who has either (1) not used at least 1 highly effective method of contraception for at least 1 month prior to screening or (2) is not willing to use such a method during treatment and for an additional 15 weeks after the end of treatment unless the subject is sterilized or postmenopausal. Menopause is defined as: 12 months of spontaneous and continuous amenorrhea in a female ≥ 55 years old or 12 months of spontaneous and continuous amenorrhea with follicle-stimulating hormone (FSH) level > 40 iU/L (according to the definition of “postmenopausal range” for the laboratory involved) in a female < 55 years old unless the subject has undergone bilateral oophorectomy. Highly effective methods of birth control include: not having intercourse or using birth control methods that work at least 99% of the time when used correctly and include: birth control pills, shots, implants, or patches, intrauterine devices (IUDs), tubal ligation/occlusion, sexual activity with a male partner who has had a vasectomy, condom or occlusive cap (diaphragm or cervical/vault caps) used with spermicide
	Subject is pregnant or breast-feeding, or planned to become pregnant during treatment and/or within 15 weeks after the end of treatment
	History or evidence of any other clinically significant disorder, condition or disease (with the exception of those outline above) that in the opinion of the Investigator or Sponsor, if consulted, would pose risk to subject safety or interferes with the study evaluation, procedures or completion (e.g. active malignancy other than squamous cell or basal cell skin cancer, use of strong or moderate CYP2C19 inhibitors, long-term concomitant treatment with non-steroidal anti-inflammatory drugs [NSAIDs])
	Unreliability as a study participant based on Investigator’s (or designee’s) knowledge of the subject (e.g. alcohol or other drug abuse, inability or unwillingness to adhere to the protocol, or psychosis)
	Patients requiring dual anti-platelet therapy at study entry
	Need for chronic oral anticoagulant therapy or chronic low-molecular-weight heparin or long-term treatment with fondaparinux
	Planned revascularization (surgical or endovascular) in any vascular territory during the duration of the study
	Planned major amputation due to PAD within the next 3 months or major amputation due to PAD within the last 30 days
	Patients who have suffered a stroke during the past 3 months
	Dementia likely to jeopardize understanding of information pertinent to study conduct or compliance to study procedures
	Known bleeding diathesis, haemostatic or coagulation disorder, or systemic bleeding, whether resolved or ongoing
	History of intracranial hemorrhage, gastrointestinal bleeding within the past 6 months, or major surgery with the past 3 months of screening (if surgical wound is judged to be associated with increased risk of bleeding and considered at risk for hemorrhagic events
	Hypersensitivity or allergic reactions to aspirin, ticagrelor, or any other products or components administered during dosing or procedures
	Concomitant use of anticoagulants such as warfarin, dabigatran, factor Xa inhibitors or antiplatelet drugs such as clopidogrel, dipyridamole and sulfapyridine
	Subject has a condition or circumstance which would prevent them from adhering to treatment regimens
	Subject has active infection (e.g. bacterial, fungal) within the previous 6 weeks prior to screening (Note: subjects with viral infection such as common cold are not excluded)
	Subject has an anemia (hemoglobin ≤ 8.5 g/dL) that requires a potential blood transfusion within 6 weeks of screening
	Subject has given blood or received a blood transfusion within the previous 3 months prior to screening
	Subject has polycythemia vera or any hyperviscosity syndrome
	Subjects with Waldenstrom’s macroglobulinemia who have an increased risk of hyperviscosity syndrome
	Subjects with known severe liver disease (e.g., ascites and/or clinical signs of coagulopathy) or obstructive liver disease [(e.g. primary biliary cirrhosis or end-stage renal disease (eGFR ≤ 30 mL/min/m^2^)]
	Subject has history of end stage renal disease (eGFR < 30 mL/min/m^2^ or renal failure requiring dialysis)
	Subject who is likely to not be available to complete all protocol-required study visits or procedures and/or to comply with all required study procedures (e.g. blood collection procedures to ensure subject safety to the best of the subject and investigator’s knowledge)
	Currently enrolled in another investigational device or drug study, or less than 30 days since ending another investigational device or drug study(s), or receiving other investigational agent(s)
	Family members or employees of the investigator or study centers involved in the study

Study participants were randomized into three groups. Each group received each of three treatments in the crossover study. Block randomization is used in block sizes of six to create groups designed to have equal and balanced sample sizes.

### Study medications

Ticagrelor and matching ticagrelor placebo were provided by AstraZeneca (Wilmington, DE). Aspirin and matching placebo were obtained from the University of Iowa Pharmaceuticals (Iowa City, IA). Ticagrelor and matching placebo, as well as aspirin and matching placebo, were compounded then over-encapsulated to maintain double-blind conditions. Stability and antimicrobial quality assurance tests were performed prior to and post over-encapsulation [22]. The Clinical Materials Services Unit of the University of Rochester Medical Center (Rochester, NY) prepared study medication kits. Non-enteric-coated aspirin 81 mg (Bayer, Inc, Pittsburgh, PA) was provided to subjects for the aspirin lead-in prior to screening and washout periods between treatment arms.

### Study procedures

#### Blood viscosity measurements

Blood viscosity is measured in vitro using a Hemathix scanning capillary viscometer (King of Prussia, PA). Whole blood samples are collected by venipuncture, anti-coagulated using ethylenediaminetetraacetic acid (EDTA) and stored between 2 and 8 °C for a maximum of 4 days prior to analysis. Temperature of blood specimens are raised to 37 °C by the viscometer for 30 min and subjected to a range of shear rates through a U-shaped capillary in a single decelerating scan for complete viscosity assessment [23, 24]. High-shear BV is reported at a shear rate of 300 s^−1^ and low-shear BV at a shear rate of 5 s^−1^.

#### Laser Doppler flowmetry

Laser Doppler flowmetry, TBI and ABI are measured using the PeriFlux 5000 (Stockholm, Sweden) as previously described [22, 25]. Both feet were examined except in the setting of unilateral amputation. Measurements were made from the hallux and the four webs and following by TBI and ABI. A sum of flow measurements was calculated for each foot.

#### Safety assessments

Study participants had measurements of the complete blood count, electrolytes and liver function tests. At the time of each study encounter, potential adverse events of special interest, including bleeding complications, were reviewed [22].

### Statistical analyses

The data were analyzed using a general linear model to account for the crossover nature of the study design, but incorporated the repeated-measure nature of the data. The variance–covariance matrix was initially assumed to have had compound symmetry, but other assumptions were considered. The main effects of the model include treatment period, sequence and the corresponding interactions. Analyses were conducted at the 5% significance level. All statistical analyses were performed using NCSS 12 Statistical Software (2018) (NCSS, LLC. Kaysville, Utah, USA, ncss.com/software/ncss).

Pre-planned contrasts comparing aspirin-ticagrelor with both ticagrelor and aspirin alone were used to evaluate the blood viscosity outcomes. These contrasts were evaluated using an experiment-wide multiple comparison procedure. Statistical considerations assumed that there was no carryover effect between treatments. In order to verify this assumption, evaluation of the treatment by period interactions was statistically tested.

The scanning capillary viscometer used in the proposed study was employed in an earlier study of 47 patients with hyperhomocysteinemia in addition to stable CVD or high CVD risk factors based on a Framingham score of > 20% [15]. In this prior CVD population, mean blood viscosity levels at a low shear rate of 5 s^−1^ were reported to be 9.98 ± 2.42 cP in the control group at baseline. A standard deviation of 2.42 cP for low-shear (5 s^−1^) blood viscosity was used throughout our sample size calculations. Separately, treatment by clopidogrel 75 mg daily for 3 weeks was reported to reduce viscosity levels by 0.6 cP and 5.0 cP at shear rates of 94.5 s^−1^ and 0.94 s^−1^, respectively [12]. Using this data, blood viscosity reduction of 2.0 cP by clopidogrel was interpolated for a shear rate of 5 s^−1^. This study was powered to detect a 2.0 cP difference in low-shear blood viscosity at a shear rate of 5 s^−1^, relative to aspirin 81 mg, with 90% power using a type I error rate of 5%. Combination aspirin-ticagrelor and low-dose aspirin control and separately, ticagrelor monotherapy and low-dose aspirin control were to be compared. This required about 72 patients in total.

The Institutional Review Board at the Icahn School of Medicine at Mount Sinai in New York, NY approved this study.

## Results

### Baseline characteristics

**Fig. 1:**
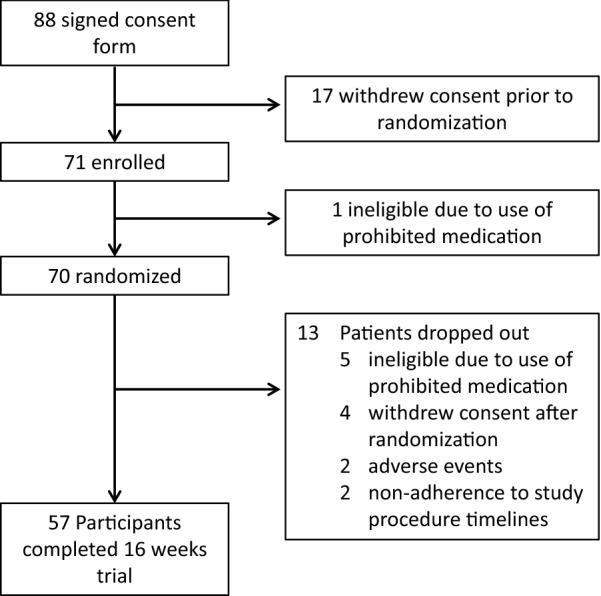
Flow of patients throughout the trial

A total of 70 participants with LEAD and type 2 diabetes were included in the final analysis population. The patient disposition throughout the trial is shown in Fig. 1. Baseline characteristics of the study population are presented in Table 2. Overall, the mean age was 72 years and 45% were women. The population was racially diverse with 58.6% Hispanic and 31.4% African American. T2DM was diagnosed for a mean 12.3 ± 10.1 years before study enrollment, 71.4% had a history of peripheral artery revascularization and 97.1% reported intermittent claudication symptoms (Additional file 1: Table S1). There was a significant burden of comorbidities in this population including 100% with diabetes including 40% on insulin, 91.4% with hypertension, 48.6% with current or former tobacco use. Most of the participants received guideline-recommended medications including 93% with statins (high-intensity 43%, moderate intensity 49%), 75% with angiotensin converting enzyme inhibitors/angiotensin receptor blockers (ACE-I/ARB) and 61% with beta-blockers. Anti-diabetic medications included 53% with metformin, 20% with sulfonylurea, 34% with DP4 inhibitor and 40% with insulin.

**Table 2:** Baseline characteristics of study participants

Age, years	70.43 ± 9
Female sex—no. (%)	34 (48.6%)
Race—no. (%)	
Hispanic	41 (58.6%)
African American	22 (31.4%)
Asian Pacific Islander	4 (5.7%)
White	3 (4.3%)
Lower extremity arterial disease	
Above the knee—no. (%)	38 (54.3%)
Below the knee—no. (%)	32 (45.7%)
Cardiovascular risk factors	
Diabetes mellitus, type 2 no. (%)	70 (100.0%)
Hypertension no. (%)	64 (91.4%)
Hyperlipidemia no. (%)	69 (98.6%)
Smoking, current or past use no. (%)	34 (48.6%)
Chronic kidney disease no. (%)	10 (14.3%)
HIV infection no (%)	22 (31.4%)
Statin use no. (%)	65 (92.9%)
High-intensity no. (%)	30 (42.9%)
Moderate-intensity no. (%)	33 (47.1%)
Low-intensity no. (%)	2 (2.9%)
Beta-blockers no. (%)	43 (61.4%)
ACE inhibitor or ARB no. (%)	52 (74.3%)
Anti-diabetic medications	
Insulin no. (%)	28 (40.0%)
Metformin no. (%)	37 (52.9%)
Sulfonylurea no. (%)	14 (20.0%)
DP4 no. (%)	24 (34.3%)
GLP agonist no. (%)	6 (8.6%)
Glitazone no. (%)	4 (5.7%)
SGLT2 no. (%)	2 (2.9%)

### Effect of trial regimen on blood viscosity

**Fig. 2:**
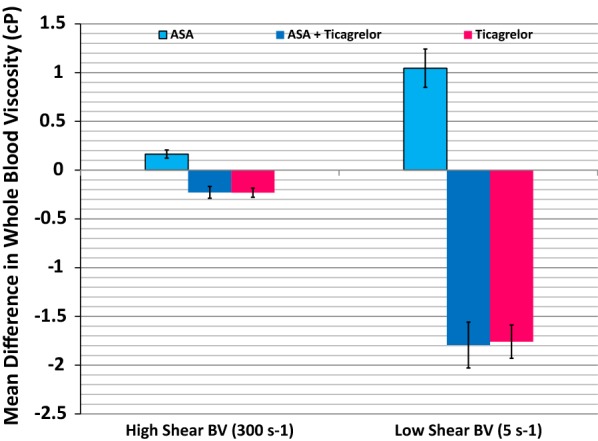
Mean levels of blood viscosity measured at low shear and high shear rate in patients treated with aspirin monotherapy, ticagrelor monotherapy and combined aspirin and ticagrelor therapy

At baseline, mean low shear BV was 11.1 ± 2.1 cP and high shear BV was 4.0 ± 0.6 cP (Table 2). When compared with aspirin monotherapy, treatment with ticagrelor either with or without aspirin reduced low shear BV by 14.2% and 13.9%, respectively, and reduced high shear BV by 5% in either case, while aspirin monotherapy increased low shear BV by 9.3% and high shear BV by 3.4% (p < 0.0001 for either high or low hear BV comparisons) (Fig. 2). There were no significant differences in BV between the groups treated with ticagrelor and ticagrelor with aspirin. There also were no significant interactions between the assignment to treatment arm and change in blood viscosity (Additional file 1: Table S2).

### Effect of trial regimen on microvascular blood flow

Combined therapy with ticagrelor and aspirin increased MBF in the left foot compared to the other two treatments (p = 0.02). Changes in MBF in the right foot were not significant (p = 0.25). The use of temperature change to assess microvascular blood vessel recruitment was not significant in either the left or right foot (Additional file 1: Table S2). In this trial, baseline blood viscosity was not associated with a change in MBF (Additional file 1: Table S2). The change in blood viscosity did not correlate with change in MBF.

The overall coefficient of variation for MBF measurements was on the order of 70%. This calculation was made on the baseline MBF measurements in the left and right feet at the time of randomization into each of the three treatment arms.

### Safety

One study participant developed dyspnea on treatment with aspirin and ticagrelor. These symptoms resolved within 24 h after discontinuing study medication. There were no major or minor hemorrhagic complications or differences in hemorrhagic complications in the treatment groups.

## Discussion

Among patients with symptomatic LEAD and long-standing type 2 diabetes, blood viscosity was lower when treated with ticagrelor alone or the combination of ticagrelor and aspirin than treatment with aspirin alone. MBF tended to be higher with combined treatment of ticagrelor and aspirin. These benefits were observed in the context of background care that included extensive use of evidence based treatments. The absolute benefit of ticagrelor with respect to the primary end point was not influenced by baseline blood viscosity.

Over the duration of this three-arm crossover trial, one patient discontinued therapy due to dyspnea. After unblinding of the trial, this subject was assigned to ticagrelor monotherapy. A total of eight participants did not complete the trial, including two patients who had urgent peripheral artery revascularization that required a prescription for P2Y12 antagonist, and six persons who were unable to complete the study schedule timelines.

The overall findings of Hema-kinesis should be placed in the context of advanced disease of the study population. Nearly three-fourths of participants had prior peripheral artery revascularization and nearly all study participants reported intermittent claudication. After peripheral artery revascularization, many patients with LEAD have repeat outpatient endovascular revascularization procedures or limb-related and cardiovascular hospitalizations within the subsequent year [26]. The most important comorbidities associated with 1-year hospitalization in the Premier Healthcare Database included diabetes, current/former smoking, hypertension and renal insufficiency. The current study includes 71.4% patients with prior revascularization who had many high-risk characteristics for MALE and cardiovascular events. Hema-kinesis selected patients with diabetes, and included 48.6% current/former smokers, 91.4% with hypertension, 31.4% with human immunodeficiency virus infection and 14.3% with renal insufficiency.

Microvascular disease is influenced by perturbations in blood rheology, endothelial dysfunction, impaired angiogenesis, hypofibrinolysis and systemic inflammation [27–29]. Although we did not measure fibrinolytic biomarkers, blood viscosity is an integrated measure of thrombosis [10]. In Hema-kinesis, blood viscosity at low shear rates was significantly reduced in ticagrelor-treated patients. Comorbidities and risk factors for LEAD have varying influence on blood viscosity and microvascular disease. Cigarette smokers have higher blood viscosity, calculated from plasma viscosity and hematocrit, than ex-smokers or former smokers. Active cigarette smoking reduced blood fluidity, an effect reversed 3 months after smoking cessation [30]. Among patients with diabetes, microvascular disease may cause neuropathy and neuropathic ulcers [27, 31] (Table 3).

**Table 3:** Baseline laboratory values of study participants

Hemorheological parameters	
Hematocrit (%)	38.3 ± 4.4
Hemoglobin—g/dL (g/L)	10.6 ± 1.4 (106 ± 14)
RBC count—M/μL	4.48 ± 0.59
WBC count—k/μL	7.1 ± 2.0
Fibrinogen—mg/dL (g/L)	351 ± 109 (3.51 ± 1.09)
Total protein (g/dL) (g/L)	7.0 ± 0.5 (70 ± 5)
Albumin (g/dL) (mmol/L)	4.2 ± 0.3 (0.63 ± 0.05)
Lipid measures	
LDL cholesterol—mg/dL (mmol/L)	83.4 ± 45.7 (2.16 ± 1.18)
Total cholesterol—mg/dL (mmol/L)	149.1 ± 50.5 (3.86 ± 1.31)
HDL cholesterol—mg/dL (mmol/L)	49 ± 17 (1.27 ± 0.44)
Non-HDL cholesterol—mg/dL (mmol/L)	104.1 ± 49.4 (2.69 ± 1.28)
Triglycerides (median [IQR])-mg/dL (mmol/L)	102 [70–152] (1.15 [0.79–1.72])
Glycemic parameters	
Fasting blood glucose—mg/dL (mmol/L)	124 ± 47 (6.88 ± 2.61)
Hemoglobin A1C—%	7.2 ± 1.1
Whole blood viscosity-cPs	
High shear	4.0 ± 0.6
Low shear	11.1 ± 2.1
Other	
Serum creatinine—mg/dL (mmol/L)	1.02 ± 0.48 (0.09 ± 0.04)
eGFR—mL/min/1.73 m^2^	54.8 ± 8.2

Elevations in blood viscosity > 4.5 cP measured at a rate of 230 s^−1^ were reported in 25–30% of patients with LEAD [25]. LEAD patients with hyperviscosity had shorter mean claudication distance and worse prognosis than claudicants with lower blood viscosity [25]. This study sought to explore the relationship between reductions in BV and MBF with ticagrelor in patients with diabetes and LEAD. An increase in MBF was observed with ticagrelor treatment. In order to explore whether there was a threshold of elevated blood viscosity that might identify higher risk subjects as proposed by Dormandy [9], we report that treatment with ticagrelor and aspirin lowered high shear blood viscosity to less than 4.5 cPs in about 50%; however, the improvement in MBF was statistically significant in the left foot and trended lower in the right foot. Changes in MBF were not different in patients stratified by tertiles of low shear or high shear blood viscosity. We acknowledge the large variability in LDF measurements may have influenced a meaningful treatment effect.

We compared the effects of ticagrelor with aspirin, an agent that has no effect on blood viscosity [24]. The reduction in blood viscosity with ticagrelor may be partially mediated by adenosine-mediated actions [16]. In an open-label study of 45 participants with coronary artery disease, the use of ticagrelor improved microvascular endothelial function as assessed by the reactive hyperemia index measured by plethysmography on the index finger of the hand. The improvement in reactive hyperemia was observed at the peak (2 h) concentration of ticagrelor and not at trough (12 h) levels. Another open-label study of 72 participants with coronary artery disease showed that 21 days treatment with ticagrelor was more effective than clopidogrel for improving microvascular endothelial function [19]. In patients with COPD undergoing coronary PCI, ticagrelor, but not clopidogrel reduced circulating epidermal growth factor levels and release of endothelial nitric oxide synthase (eNOS) from cultured human umbilical vein endothelial cells [32]. The authors suggest that by lowering EGF levels, ticagrelor improves endothelial function by activation of eNOS.

Anti-platelet therapy has been considered a mainstay of treatment for LEAD [33, 34]. The Examining Use of Ticagrelor in Peripheral Artery Disease (EUCLID) trial reported no difference in cardiovascular outcomes or acute ischemic limb events in patients without and with diabetes. In EUCLID, the cardiovascular event rate was higher in the 38.5% of patients with versus without diabetes 15.9% versus 10.4%, adjusted hazard ratio 1.56 [95% CI 1.41 to 1.72] [35]. Subsequently, THEMIS (The Effect of Ticagrelor on Health Outcomes in diabEtes Mellitus patients Intervention Study) investigated the efficacy of aspirin and ticagrelor 60 mg twice daily versus aspirin alone on major adverse cardiovascular events in patients with coronary artery disease and type 2 diabetes who had no prior myocardial infarction or stroke [36]. In a press announcement, the trial sponsor reported a significant reduction in major adverse cardiovascular events (Brilinta reduced cardiovascular events in patients with no prior heart attack or stroke. Press release AstraZeneca 25 February 2019 07:00 GMT). The use of vorapaxar, a protease-activated receptor antagonist, added to background treatment with aspirin, P2Y_12_ inhibitors or both, reduced acute limb ischemia and limb revascularization [37]. Low-dose rivaroxaban and aspirin was more effective than aspirin alone for reducing major adverse limb events including ischemia within 30 days or ischemic amputation [38].

### Strengths and limitations

Several strengths of this trial include the use of a randomized, double-blind, double-dummy, controlled crossover trial design in which all study participants received each treatment. A crossover design was a crucial aspect of mechanistic trial that eliminates differences in patient characteristics. Due to the effects of cigarette smoking on blood viscosity and blood fluidity, none of the study participants changed their smoking behavior during the course of the trial. Limitations of a crossover trial requires a longer time commitment from study participants. During the course of the trial, two participants required urgent revascularization for ischemic ulcers. A total of 12 participants did not meet study timelines, and were not exposed to all three treatments. Microvascular blood flow was measured by laser Doppler flowmetry; however, we found this method highly variable. The variability in microvascular blood flow measurements reduced the ability to consistently ascertain the effects of blood viscosity reductions on this functional parameter. Mahe et al. reported a coefficient of variation of 40% for the local thermal hyperemia peaks using the laser speckle contrast imager and LDF.

## Conclusions

This trial supports the use of targeted anti-platelet therapy with ticagrelor in patients with LEAD. Further research is needed to investigate the contribution of ticagrelor as therapy for the prevention of microvascular complications of diabetes. More robust methods for the assessment of microvascular blood flow in the lower extremities are needed for evaluation of microvascular complications.

## Additional file

**Additional file 1: Table S1.** Characteristics of peripheral artery disease in study participants. **Table S2.** Results of ABI, TBI and LBF in study participants.

## Data Availability

The data are available for review by external regulatory agencies by prior written consent.

## Abbreviations

ABI: ankle brachial index
BV: blood viscosity
ENT1: equilibrative nucleoside transporter 1
LDF: laser Doppler flowmetry
LEAD: lower extremity arterial disease
MALE: major adverse limb events
MBF: microvascular blood flow
T2DM: type 2 diabetes mellitus
TBI: toe-brachial index

## References

[1] Fowkes FG, Rudan D, Rudan I, Aboyans V, Denenberg JO, McDermott MM, Norman PE, Sampson UK, Williams LJ, Mensah GA (2013). Comparison of global estimates of prevalence and risk factors for peripheral artery disease in 2000 and 2010: a systematic review and analysis. Lancet.

[2] Sampson UK, Fowkes FG, McDermott MM, Criqui MH, Aboyans V, Norman PE, Forouzanfar MH, Naghavi M, Song Y, Harrell FE (2014). Global and regional burden of death and disability from peripheral artery disease: 21 world regions, 1990 to 2010. Glob Heart.

[3] Luscher TF, Creager MA, Beckman JA, Cosentino F (2003). Diabetes and vascular disease: pathophysiology, clinical consequences, and medical therapy: part II. Circulation.

[4] Selvin E, Erlinger TP (2004). Prevalence of and risk factors for peripheral arterial disease in the United States: results from the National Health and Nutrition Examination Survey, 1999-2000. Circulation.

[5] Schneider F, Saulnier PJ, Gand E, Desvergnes M, Lefort N, Thorin E, Thorin-Trescases N, Mohammedi K, Ragot S, Ricco JB (2018). Influence of micro- and macro-vascular disease and Tumor Necrosis Factor Receptor 1 on the level of lower-extremity amputation in patients with type 2 diabetes. Cardiovasc Diabetol.

[6] Huang D, Refaat M, Mohammedi K, Jayyousi A, Al Suwaidi J, Abi Khalil C (2017). Macrovascular complications in patients with diabetes and prediabetes. Biomed Res Int.

[7] Cho YI, Mooney MP, Cho DJ (2008). Hemorheological disorders in diabetes mellitus. J Diabetes Sci Technol.

[8] Dormandy JA, Hoare E, Postlethwaite J (1974). Importance of blood viscosity. Rheological claudication. Proc R Soc Med.

[9] Dormandy J, Hoare E, Postlethwaite J (1976). Blood viscosity of patients with intermittent claudication—concept of “rheological claudication”. Biorheology.

[10] Cowan AQ, Cho DJ, Rosenson RS (2012). Importance of blood rheology in the pathophysiology of atherothrombosis. Cardiovasc Drugs Ther.

[11] Cho YI, Cho DJ, Rosenson RS (2014). Endothelial shear stress and blood viscosity in peripheral arterial disease. Curr Atheroscler Rep.

[12] Ciuffetti G, Lombardini R, Pirro M, Lupattelli G, Mannarino E (2001). Clopidogrel: hemorheological effects in subjects with subclinical atherosclerosis. Clin Hemorheol Microcirc.

[13] Solerte SB, Fioravanti M, Cerutti N, Severgnini S, Locatelli M, Pezza N, Rondanelli M, Trecate L, Balza G, Ferrari E (1997). Retrospective analysis of long-term hemorheologic effects of pentoxifylline in diabetic patients with angiopathic complications. Acta Diabetol.

[14] Perego MA, Sergio G, Artale F, Giunti P, Danese C (1986). Haemorrheological improvement by pentoxifylline in patients with peripheral arterial occlusive disease. Curr Med Res Opin.

[15] Dawson DL, Zheng Q, Worthy SA, Charles B, Bradley DV (2002). Failure of pentoxifylline or cilostazol to improve blood and plasma viscosity, fibrinogen, and erythrocyte deformability in claudication. Angiology.

[16] Bonello L, Laine M, Kipson N, Mancini J, Helal O, Fromonot J, Gariboldi V, Condo J, Thuny F, Frere C (2014). Ticagrelor increases adenosine plasma concentration in patients with an acute coronary syndrome. J Am Coll Cardiol.

[17] Armstrong D, Summers C, Ewart L, Nylander S, Sidaway JE, van Giezen JJ (2014). Characterization of the adenosine pharmacology of ticagrelor reveals therapeutically relevant inhibition of equilibrative nucleoside transporter 1. J Cardiovasc Pharmacol Ther.

[18] Ohman J, Kudira R, Albinsson S, Olde B, Erlinge D (2012). Ticagrelor induces adenosine triphosphate release from human red blood cells. Biochem Biophys Res Commun.

[19] Alemayehu M, Kim RB, Lavi R, Gong I, D’Alfonso S, Mansell SE, Wall S, Lavi S (2017). Effect of ticagrelor versus clopidogrel on vascular reactivity. J Am Coll Cardiol.

[20] Wittfeldt A, Emanuelsson H, Brandrup-Wognsen G, van Giezen JJ, Jonasson J, Nylander S, Gan LM (2013). Ticagrelor enhances adenosine-induced coronary vasodilatory responses in humans. J Am Coll Cardiol.

[21] Rosenson RS (2008). Treatment with aspirin and dipyridamole is more effective than aspirin in reducing low shear blood viscosity. Microcirculation.

[22] Rosenson RS, Chen Q, Najera SD, Lee ML, Cho DJ (2018). Ticagrelor and the prevention of microvascular complications in diabetes patients with lower extremity arterial disease; rationale and design of the Hema-kinesis trial. Cardiovasc Drugs Ther.

[23] Wang S, Boss AH, Kensey KR, Rosenson RS (2003). Variations of whole blood viscosity using Rheolog-a new scanning capillary viscometer. Clin Chim Acta Int J Clin Chem.

[24] Rosenson RS, Wolff D, Green D, Boss AH, Kensey KR (2004). Aspirin. Aspirin does not alter native blood viscosity. J Thromb Haemost JTH.

[25] Chen Q, Rosenson RS (2018). Systematic review of methods used for the microvascular assessment of peripheral arterial disease. Cardiovasc Drugs Ther.

[26] Hess CN, Rogers RK, Wang TY, Fu R, Gundrum J, Allen lapointe NM, Hiatt WR (2018). Major adverse limb events and 1-year outcomes after peripheral artery revascularization. J Am Coll Cardiol.

[27] Hamburg NM, Creager MA (2017). Pathophysiology of intermittent claudication in peripheral artery disease. Circ J.

[28] Beckman JA, Creager MA (2016). Vascular complications of diabetes. Circ Res.

[29] Kearney K, Tomlinson D, Smith K, Ajjan R (2017). Hypofibrinolysis in diabetes: a therapeutic target for the reduction of cardiovascular risk. Cardiovasc Diabetol.

[30] Shimada S, Hasegawa K, Wada H, Terashima S, Satoh-Asahara N, Yamakage H, Kitaoka S, Akao M, Shimatsu A, Takahashi Y (2011). High blood viscosity is closely associated with cigarette smoking and markedly reduced by smoking cessation. Circ J.

[31] Wannamethee SG, Lowe GD, Shaper AG, Rumley A, Lennon L, Whincup PH (2005). Associations between cigarette smoking, pipe/cigar smoking, and smoking cessation, and haemostatic and inflammatory markers for cardiovascular disease. Eur Heart J.

[32] Vieceli Dalla Sega F, Fortini F, Aquila G, Pavasini R, Biscaglia S, Bernucci D, Del Franco A, Tonet E, Rizzo P, Ferrari R (2018). Ticagrelor improves endothelial function by decreasing circulating epidermal growth factor (EGF). Front Physiol.

[33] Bhatt DL, Flather MD, Hacke W, Berger PB, Black HR, Boden WE, Cacoub P, Cohen EA, Creager MA, Easton JD (2007). Patients with prior myocardial infarction, stroke, or symptomatic peripheral arterial disease in the CHARISMA trial. J Am Coll Cardiol.

[34] Gerhard-Herman MD, Gornik HL, Barrett C, Barshes NR, Corriere MA, Drachman DE, Fleisher LA, Fowkes FG, Hamburg NM, Kinlay S (2017). 2016 AHA/ACC guideline on the management of patients with lower extremity peripheral artery disease: a report of the american college of cardiology/american heart association task force on clinical practice guidelines. Circulation.

[35] Hiatt WR, Fowkes FG, Heizer G, Berger JS, Baumgartner I, Held P, Katona BG, Mahaffey KW, Norgren L, Jones WS (2017). Ticagrelor versus clopidogrel in symptomatic peripheral artery disease. N Engl J Med.

[36] Bhatt DL, Fox K, Harrington RA, Leiter LA, Mehta SR, Simon T, Andersson M, Himmelmann A, Ridderstrale W, Held C (2019). Rationale, design and baseline characteristics of the effect of ticagrelor on health outcomes in diabetes mellitus patients Intervention study. Clin Cardiol.

[37] Bonaca MP, Gutierrez JA, Creager MA, Scirica BM, Olin J, Murphy SA, Braunwald E, Morrow DA (2016). Acute limb ischemia and outcomes with vorapaxar in patients with peripheral artery disease: results from the trial to assess the effects of vorapaxar in preventing heart attack and stroke in patients with atherosclerosis-thrombolysis in myocardial infarction 50 (TRA2 degrees P-TIMI 50). Circulation.

[38] Anand SS, Bosch J, Eikelboom JW, Connolly SJ, Diaz R, Widimsky P, Aboyans V, Alings M, Kakkar AK, Keltai K (2018). Rivaroxaban with or without aspirin in patients with stable peripheral or carotid artery disease: an international, randomised, double-blind, placebo-controlled trial. Lancet.

