# Supplementary material for: Ticagrelor improves blood viscosity-dependent microcirculatory flow in patients with lower extremity arterial disease: the Hema-kinesis clinical trial
Source: Cardiovasc Diabetol. 2019 Jun 7;18:77. doi: 10.1186/s12933-019-0882-5 (PMC6556022; doi:10.1186/s12933-019-0882-5)
Supplement: Supplementary file 1 — Additional file 1: Table S1. Characteristics of peripheral artery disease in study participants. Table S2. Results of ABI, TBI and LBF in study participants. [file 12933_2019_882_MOESM1_ESM.docx]

Table S1: Characteristics of peripheral artery disease in study participants.

|  | Incidence (%) | Average duration |
| --- | --- | --- |
| History of Diabetes | 100 | 12.3±10.1 |
| Intermittent claudication | 97.1 | N/A |
| Single vessel disease | 7.1 | N/A |
| Multi vessel disease | 92.9 | N/A |
| Unilateral | 32.9 | N/A |
| Bilateral | 67.1 | N/A |
| History of revasculatization | 71.4 | N/A |

Table S2: Results of ABI, TBI and LBF in study participants.

|  | Right | Left |
| --- | --- | --- |
| ABI |  |  |
| ASA | 0.038±0.013 | 0.015±0.067 |
| Ticagrelor | 0.095±0.051 | 0.075±0.029 |
| Both | 0.021±0.019 | 0.032±0.038 |
| TBI |  |  |
| ASA | 0.044±0.054 | 0.004±0.022 |
| Ticagrelor | 0.011±0.007 | 0.036±0.028 |
| Both | -0.009±0.015 | -0.016±0.017 |
| LBF |  |  |
| ASA | -4.10±13.30 | -5.60±11.86 |
| Ticagrelor | -5.23±7.76 | -4.28±7.48 |
| Both | 13.65±7.34 | 24.76±8.36 |
